# Antenatal and Postpartum Depression: Prevalence and Associated Risk Factors among Adolescents' in KwaZulu-Natal, South Africa

**DOI:** 10.1155/2020/5364521

**Published:** 2020-01-21

**Authors:** Desiree Govender, Saloshni Naidoo, Myra Taylor

**Affiliations:** ^1^KwaZulu-Natal Department of Health, South Africa; ^2^School of Nursing and Public Health, Discipline of Public Health Medicine, University of KwaZulu-Natal, South Africa; ^3^Developing Research Innovation Localisation and Leadership (DRILL) Fellow, South Africa

## Abstract

**Background:**

Maternal depression is a major public health concern as it affects both mothers and their children. Antenatal depression, which is often underdiagnosed, has been associated with preterm labour, low birth weight, and intrauterine growth restriction. Research has demonstrated that postpartum depression is associated with mother-infant bonding impairment, child abuse, child neglect, maternal substance abuse, and self-harm. Globally, the prevalence of depression in pregnant and postpartum adolescents varies. This paper reports on the findings of the prevalence of depression and its associated risk factors among pregnant and postpartum adolescents in KwaZulu-Natal, South Africa.

**Methods:**

Data were generated by means of a descriptive cross-sectional study that was conducted between June and November 2017 utilizing a sample of 326 adolescent females accessing maternal health services in a medium-sized rural peripheral district hospital in Ugu, Southern KwaZulu-Natal. The Edinburgh Postnatal Depression Scale questionnaire was used to screen participating pregnant and postnatal adolescents for depression. A cut-off score of ≥13 was used to identify pregnant and postnatal adolescents with symptoms of depression. The data were analysed using R software.

**Results:**

The prevalence of depression among the pregnant participants was 15.9% (21/132), whereas it was 8.8% (17/194) among the postpartum participants. Antenatal depression was associated with physical violence (adjusted odds ratio (aOR) 6.47, 95% CI 1.36-30.53, *p* = 0.01) and verbal abuse (adjusted odds ratio (aOR) 4.8, 95% CI 1.5-15.16, *p* = 0.006). The pregnant participants who indicated they received a lot of support from their partners were 0.93% less likely to have depression. Postnatal depression was associated with physical violence (adjusted odds ratio (aOR) 7.32, 95% CI 1.66-29.44, *p* = 0.005), verbal abuse (adjusted odds ratio (aOR) 4.3, 95% CI 1.03-15.79, *p* = 0.03), and intimate partner violence (adjusted odds ratio (aOR) 9.58, 95% CI 1.58-48.82, *p* = 0.008).

**Conclusion:**

The prevalence of antenatal depression was higher than postpartum depression in the study sample. In light of the findings, maternal healthcare professionals are cautioned to consider the mental health of pregnant and postpartum adolescents who seek their services at health facilities.

## 1. Introduction

Depression during pregnancy and in the postpartum period is a major public health concern as it affects both mothers and their babies [1]. Antenatal depression, which is often underdiagnosed, has been associated with preterm labour [2], spontaneous abortion, [3] low birth weight [4], and intrauterine growth restriction [5]. Research has demonstrated that postpartum depression is associated with mother-infant bonding impairment, child abuse, child neglect, maternal substance abuse, and self-harm [6, 7]. Maternal depression has also been linked to poor weight gain and impaired cognitive and motor development in infants [6]. Postpartum depression has been described as “a thief that steals motherhood” [8], particularly as depressed mothers may prematurely discontinue breastfeeding due to the reduction of breast milk production six months postpartum [6]. Moreover, the depressed state of mothers can also induce depression in infants [9].

Pregnant and postpartum adolescents are more likely to suffer from depression than their nonpregnant counterparts [10]. Moreover, the lives of pregnant and postpartum adolescents differ from their nonpregnant counterparts. Pregnant and postpartum adolescents are more likely to have higher rates of physical and sexual abuse, exposure to community violence, and poor access to healthcare services [10–12]. Early childbearing increases the risk of adverse mental health problems (depression, substance abuse, and posttraumatic stress disorder) [11], and it has been found that adolescent mothers have a higher risk of depression than adolescent fathers. [10]. Furthermore, adolescent pregnancy and motherhood is associated with stigma, discrimination, gender inequalities, and disruption of educational goals [12].

The risk factors associated with clinical depression among pregnant and postpartum adolescents include dysfunctional family structures, low socioeconomic status, lack of family support, social isolation, history of physical and sexual abuse, partner neglect, and elevated stress levels [13, 14]. The discrimination against and stereotyping of adolescent mothers' lead to social exclusion and a high risk for postpartum depression [15, 16]. Adolescent mothers have to juggle many roles such as raising a child, finishing school, and finding employment [14]. South African adolescent mothers have also reported experiencing conflict between the role of being a mother and that of being an adolescent [17], and it is thus undeniable that the transition to parenthood for an adolescent is extremely stressful [16].

The perception and experience of motherhood are different in every culture. According to Abdollahi et al. [18], “the diversity of the prevalence of postpartum depression across the cultures may assist researchers in understanding whether this disorder is primarily brought on by psychological or biological factors.” In the South African context, childbearing in women is valued [19]. However, in the event of unplanned pregnancies, rejection of the pregnancy by the partner and a lack of family support for the pregnant and parenting adolescent can be emotionally distressing [19]. It is not surprising that antenatal and postnatal depression will be evident under these circumstances. Approximately, seventy-one percent of pregnancies among 15–19 year olds in South Africa are unplanned [20]. These unplanned pregnancies can subsequently lead to psychological distress, anxiety, and depression. Cultural stigma, social stigma, oppressive familial dynamics, and gender inequality impact on the mental health of pregnant and postpartum adolescents [20].

Globally, the prevalence of depression in pregnant and postpartum adolescents varies [21]. It has been found that the prevalence of maternal depression is higher in low and middle income countries than in high income countries [22] and that adolescent mothers may experience higher rates of prenatal and postnatal depression than adult mothers [23]. Approximately 20% of adolescents' experience symptoms of depression during pregnancy as well as postpartum [23]. However, the accurate figure of depression among pregnant and postpartum adolescents may be higher because many are not screened for symptoms of depression or are unaware of prenatal and postpartum depression [23]. Against this backdrop, pregnant and postpartum adolescents' may suffer in silence. As knowledge of the prevalence of depression and its associated risk factors among pregnant and postpartum adolescents in South Africa is still vague, an investigation into this phenomenon was conducted among pregnant and postpartum adolescents in Ugu district, Southern KwaZulu-Natal.

## 2. Materials and Methods

### 2.1. Data Source

Data were drawn from a quantitative survey of a larger PhD study that evaluated adolescent pregnancy and sexual and reproductive health in order to inform the development of a community of practice model for multidisciplinary and comprehensive care of pregnant and parenting adolescents. The adolescent pregnancy, sexual, and reproductive health questionnaire survey was designed to elicit information regarding adolescents' social, obstetric, and clinical demographics; their knowledge of pregnancy, sexual, and reproductive health; their partner relationship characteristics; symptoms of depression; and their health practices during pregnancy. The questionnaire was developed in consultation with maternal health clinicians, a school health manager, and clinical psychologists. The formulation of questions on sociodemographic characteristics was guided by an ecological perspective on adolescent pregnancy. The survey sample comprised adolescent females aged 13 to 19 years.

### 2.2. Study Design and Setting

This descriptive cross-sectional study was conducted between June and November 2017 utilizing a convenience sample of 326 adolescent females who had accessed maternal health services in a medium-sized (300 bed) rural peripheral district hospital in Ugu, KwaZulu-Natal. Details of the sample size calculation have been reported earlier [24]. The sample size of 326 participants was determined for a study on the prevalence of adolescent repeat pregnancy [24]. The following quantities that were required to calculate the sample size included the anticipated proportion (expected prevalence), the confidence interval, and the desired precision (acceptable margin of error). The absolute error or precision was 5% with an infinite population size, a 95% confidence interval, and an anticipated proportion of 17.6% which yielded a minimum sample size of 223. We obtained a sample size of 326 after adjusting for a nonresponse rate of 45%.

Ugu health district is 84% rural and 16% urban. The unemployment rate is estimated at 27.4% of an estimated population of 753 336. Children and the youth make up 38.7% of the total population. At the time of the study, 65% of the youth in Ugu district have completed matric (final year of high school) but only 4% have attained tertiary (education pursued beyond the high school level) qualifications. Approximately 66.5% of the population survives on social grants (grants paid by government to South African citizens who are in need of assistance). The poverty index in Ugu district is 42% [25].

### 2.3. Measurement Instrument

The Edinburgh Postnatal Depression Scale (EPDS) was used to assess antenatal and postpartum depression in this study. Although the EPDS was developed to screen for postpartum depression, many studies have validated and used the EPDS in pregnancy surveys [22, 26–31]. Research in both urban and rural areas of South Africa has shown that the EPDS is a validated screening instrument [32–36], whereas other sub-Saharan countries have also validated the EPDS to screen for depression during and after pregnancy [19, 32–35]. The EPDS comprises ten self-report items that evaluate depressive symptoms in the past seven days. There are four possible responses for each of the questions which adds to a total score of 30. The EPDS is a preferred tool because it excludes somatic symptoms that could coincide with the normal physiological changes associated with pregnancy and thus confound the assessment [30]. According to a systematic review by Nast et al. [31] of psychometric instruments to assess psychosocial stress during pregnancy, the EPDS “is the best available instrument for measuring depressive symptomatology during pregnancy.” The EPDS displayed high reliability and validity coefficients in pregnancy populations. These somatic symptoms include changes in appetite, fatigue, and sleep disturbances that are also associated with depression. The EPDS focuses on the cognitive and behavioural symptoms of depression.

The strength of the EPDS also includes the prediction of postpartum depression in the immediate postdelivery period [9, 37]. A study in Canada found that when various tools were administered 48 to 72 hours (2-3 days) postdelivery, the EPDS was the only tool that was able to accurately predict scores in the 4-6-week postpartum period [38]. The validated cut-off score is ≥13 to screen positively for depression.

### 2.4. Data Collection

Data were collected by trained fieldworkers from the local community using mobile devices powered by the Mobenzi Researcher® technology. The data collection procedures and the features of Mobenzi Researcher® have been described previously [24]. Mobenzi Researcher® is a mobile data collection tool. The innovative technology of Mobenzi Researcher® allows surveys to be deployed through the mobile device. The mobile-assisted face-to-face interviews between the fieldworkers and individual female adolescents were captured on the mobile device. The data on the mobile device were uploaded to the Mobenzi server, stored, and aggregated on an Excel spreadsheet.

### 2.5. Data Analysis

The data from the Mobenzi server were cleaned and exported into R software (R version 3.5.0. Vienna: R Foundation for Statistical Computing) for statistical analyses. Frequency distributions and summary statistics were generated to describe the data. Bivariate and logistic regression analyses were conducted to determine the relationship between depression (score of 13 and above on the EPDS) and selected variables. Univariable and multivariable models were produced. A *p* value of 0.05 was considered statistically significant.

### 2.6. Ethical Considerations

The study was approved by the Biomedical Research Ethics Committee of the University of KwaZulu-Natal (BE553/16) and the Department of Health, KwaZulu-Natal (ref no: KZ_2016RP26_545). Institutional approval was obtained from GJ Crookes Hospital to conduct the study among patients. All the participants provided informed consent or assent prior to data collection. Parents' or legal guardians' permission was obtained for participants under the age of 18 who agreed to participate in this study.

## 3. Results

### 3.1. Social, Obstetric, and Clinical Demographics of the Participants

The median age of the participants was 18 (IQR 17-19 years), and the median number of people contributing to the household income was 2 (IQR 1-3) (Table 1). Of the 326 participants, 13.5% are between the ages of 14 and 16 years. The majority of the participants were in the postpartum period (*n* = 194, 59.5%) (Table 2). The EPDS was administered to these participants 1 week postpartum. Only nine (2.8%) of the participants reported that their highest education level was primary school, while 93.6% had a secondary school education. Of the 326 participants, 318 (97.5%) were unemployed. Less than half of the participants' mothers were single (*n* = 147, 45.1%). The prevalence of spontaneous abortion was 8.6% (*n* = 28). The prevalence of adolescent repeat pregnancy was 19.9% (*n* = 65) while HIV (known status) prevalence was 17.8% (*n* = 58). The desire to incur self-harm was reported by 9.8% (*n* = 32) of the participants. Reports of physical, sexual, and verbal abuse were 6.1% (*n* = 20), 3.7% (*n* = 12), and 10.4% (*n* = 34), respectively, while the prevalence of intimate partner violence (IPV) was 3.7% (*n* = 12).

Of the sample of 326 participants, 11.7% (*n* = 38) had a score of 13 and higher on the Edinburgh Postnatal Depression Scale, indicating probable depression among less than 15% of the sample. However, a higher frequency of depression was revealed for pregnant than for postpartum participants (Table 3). The gestational age of the pregnant participants were 24 weeks and more. Of the total of 38 participants with probable depression, 21 (55.3%) were pregnant while 17 (44.7%) were in the postpartum period. The prevalence of depression among the pregnant participants was 15.9% (21/132) whereas the prevalence of depression among the postpartum participants was 8.8% (17/194).

### 3.2. Bivariate Analyses

The bivariate analyses revealed that the experiences of physical, sexual, and verbal abuse were associated with the risk of depression (*p* = 0.0007, *p* = 0.010, and *p* = 0.002, respectively) (Table 4). The depressed participants were more likely to receive no support from their partners than the nondepressed participants.

Risk factors of depression in the pregnancy period were also determined (Table 5), and it was found that both physical abuse (*p* = 0.036) and verbal abuse (*p* = 0.007) were associated with the risk of depression during the pregnancy period. Moreover, the pregnant and depressed participants were more likely to indicate no partner support. Among the postpartum participants, experiences of physical abuse (*p* = 0.009), verbal abuse (*p* = 0.049), and intimate partner violence (*p* = 0.013) were associated with the risk of depression (Table 6). Postpartum and depressed participants were more likely to have only a primary education than those who were postpartum and not depressed (*p* = 0.077).

### 3.3. Logistic Regression Models

Models were constructed using logistic regression. In each model, there was an outcome variable (depression) regressed upon a primary “exposure of interest” variable. The exposure variables were chosen based upon variables that had significant differences between depression groups in the bivariate analyses: i.e., the experience of physical, sexual, and verbal abuse; intimate partner violence; and support from partner. These models were stratified by whether or not the adolescent was pregnant or postpartum. For each model, an odds ratio (OR) for each of the exposure of interest in the model and a 95% confidence interval were produced.

Finally, for the models containing sexual abuse, verbal abuse, and intimate partner violence, the third categories were removed because there were so few observations in those categories. Tables 7 and 8 present the results of both univariable and multivariable models. The multivariable model adjusts for age, HIV status, and the number of people in the household contributing to income. These three variables are candidates for confounding because they are related to both the primary exposures and the outcome (depression), and do not seem to be on the causal pathway between exposure and outcome (i.e., they are likely not mediators).

### 3.4. Factors Associated with Antenatal Depression

In the unadjusted model, those who were pregnant and experienced physical violence were 4.99 times more likely to have depression (Table 7). When controlling for confounding variables (age, number contributing to household income, and HIV), that association strengthened. The pregnant participants were actually 6.47 times more likely to have depression than those who had not experienced physical violence.

Sexual abuse was not associated with antenatal depression, even after controlling for the confounding variables (Table 7). In the unadjusted model, those who were pregnant and had experienced verbal abuse were 5 times more likely to have depression. When controlling for confounding variables, that association slightly weakened. They were actually only 4.82 times more likely to have depression than those who had not experienced physical violence. Intimate partner violence does not seem to have a relationship with antenatal depression, even after controlling for the confounding variables. Pregnant adolescents who indicated that they had a lot of support from their partners were 0.83% less likely to have depression compared with those pregnant adolescents who had indicated they had no partner support. After adjusting for the confounders, the relationship between partner support and antenatal depression strengthened. The pregnant adolescents with a lot of support were 0.93% less likely to have depression.

### 3.5. Factors Associated with Postpartum Depression

After controlling for confounding variables, postpartum adolescents experiencing physical violence were 7.32 times more likely to have depression than those not experiencing physical violence (the aOR was 6.47 between physical violence and depression during pregnancy) (Table 8). There was no relationship between sexual abuse and postpartum depression. After adjusting for confounding variables, the association between verbal abuse and postpartum depression increased (adjusted odds ratio (aOR) 4.3, 95% CI 1.03-15.79, *p* = 0.03). Intimate partner violence was strongly associated with postpartum depression. After controlling for confounding variables, postpartum adolescents experiencing intimate partner violence were 9.18 times more likely to have postpartum depression (Table 8).

## 4. Discussion

Global estimates of antenatal and postpartum depression are 15.6% and 19.8%, respectively, in low and middle income countries [39]. Our study found that the prevalence of antenatal depression was 15.9% among the pregnant participants whereas it was 8.8% among the postpartum participants. Many research studies have focused on the prevalence and risk factors regarding the antenatal and postpartum depression in adult women of reproductive age. However, few studies have examined the prevalence of depression and associated risk factors among pregnant and parenting adolescent women. In this regard, the comparisons in our discussion also refers to studies of antenatal and postpartum depression in adult women of reproductive age due to limited studies of adolescents in this area.

The prevalence of antenatal depression in China (participants aged 20 to 35 years) and coastal South India (participants aged 19 to 44 years) was very similar at 13.7% and 16.3%, respectively [40, 41]. However, the prevalence of 15.9% for antenatal (during pregnancy) depression that was observed for our sample of participants was lower than the prevalence of 38.5% reported in a study in Durban, KwaZulu-Natal [34]. The age range of participants in the Durban study was 14 to 46 years. The reported prevalence rates of antenatal depression in other African countries were 24.5%, 21%, and 25% in Nigeria (participants aged 15 to 49 years), Malawi (participants aged 20 to 29 years), and Ethiopia, respectively [26, 42, 43]. Adolescents made up 8.7% of the 393 participants who were screened for antenatal depression in Ethiopia.

The prevalence of postpartum depression at 8.8% that was observed for our sample of participants was comparable to the prevalence of 7% that was observed in a study conducted in Ghana (participants aged 18 to 51 years and above) [44]. The prevalence of postpartum depression among a sample of nine hundred and eleven postpartum adolescents in a study in Kolkata, India, was 34.1% [45]. A study in the Witzenberg subdistrict in the Western Cape in South Africa found that the prevalence of postpartum depression was 50.3% [34], while study in South-West Ethiopia reported a prevalence of 33.8% [22]. Adolescents comprised of 23.9% of the participants in the Witzenberg, Western Cape study whereas participants in Ethiopia were in the age range of 15-24 years with mean age of 26.06 years.

Previous studies on antenatal and postpartum depression in South Africa and other sub-Saharan Africa studies included adolescent and adult women as participants [27, 33, 34, 42, 43]. The current study found a lower prevalence of antenatal and postpartum depression among the participants compared to previous South African and other sub-Saharan studies [27, 33, 34, 42, 43]. There are several possible explanations for this result. The differences in the prevalence rates between the current study and previous local studies could be attributed to the sociodemographic, cultural, and economic characteristics of the participants as well as to geographical settings (urban versus rural) and the study design (observation period, sample size, screening, and methods versus diagnostics). It is also important to note that society views mental illness as a detriment to ideal motherhood [9], which may have resulted in the underreporting of the psychological distress and struggles that the mothers may have experienced [9]. The stigma associated with maternal mental illness may also have caused the women, particularly as they were adolescent girls, to feel ashamed as they may have feared being labeled as unfit mothers [46].

The literature identifies maternal age, socioeconomic status, unplanned pregnancies, violence, social support, history of previous mental disorder, social support, and pregnancy-related complications as risk factors for maternal depression [13]. In this study, antenatal depression was associated with physical violence (adjusted odds ratio (aOR) 6.47, 95% CI 1.36-30.53, *p* = 0.03) and verbal abuse (adjusted odds ratio (aOR) 4.8, 95% CI 1.5-15.16, *p* = 0.006). Partner support was found to be a protective factor against depression during pregnancy (adjusted odds ratio (aOR) 0.07, 95% CI 0.01-0.45, *p* = 0.005). A study that was conducted in Australia identified the following similar risk factor rates for antenatal depression: absence of partner support (odds ratio (OR) 6.1, 95% CI 4.6-7.9, *p* < 0.001), physical intimate partner violence (odds ratio (OR) 2.6, 95% CI 1.6-4.2, *p* < 0.001), and psychological intimate partner violence (odds ratio (OR) 4.8, 95% CI 3.0-7.8, *p* < 0.001) [43]. Of the 243 adolescents who participated in the Australian study, 9.9% were diagnosed with antenatal depression. It has often been demonstrated that intimate partner violence can endanger the life of the mother and her unborn child, and thus, screening for intimate partner violence is important in the antenatal and postpartum periods [27].

The transition into motherhood can be complex and stressful [16]. A supportive social network (family, partner, and friends) may help ease the stress of pregnancy and motherhood [15], particularly for young adolescent girls. However, limited social support as a significant risk factor was also identified in Nairobi, Kenya (*p* = 0.008) [47, 48]. The study population in Kenya included pregnant adolescent women 15 to 18 years of age. Unfortunately, pregnant and postpartum adolescents are likely to have poor support as most of them are single and not living with their partners [49]. The stigma regarding early and premarital childbearing also poses a hindrance to adolescent mothers who require social support [15]. Many find interpersonal relationships difficult, and they are often unable to relate to peers who have not experienced pregnancy and motherhood [49]. Social isolation is commonly reported by adolescent mothers, and depression thrives in the face of social isolation [49].

Depression and anxiety are more prevalent among HIV-infected pregnant women. The stigma attached to HIV/AIDS, concerns of the transmission of the virus to the fetus, and the denial of its existence exacerbate the stress pregnant women experience [50]. HIV infection has a major impact on mental health. The prevalence of HIV among South African pregnant adolescents and young women in the age group 15-24 years was 19.2% in 2015 [51]. According to the Lancet Maternal Health series, 2014, many African women learn of their HIV diagnosis during pregnancy, and this increases their susceptibility to depression [52].

In the current study, HIV seropositivity was not associated with maternal depression, which seem to be consistent with the findings of a study conducted in two hospitals in KwaZulu-Natal by Nydoo et al. [50]. The maternal age of the sample ranged from 17 to 40 years in the study conducted by Nydoo et al. [50]. It is interesting to note that previous studies conducted in Durban, South Africa [34], and Nairobi, Kenya [48], have demonstrated a statistically significant relationship between HIV status and maternal depression. A study in Nigeria found that young maternal age (*p* = 0.014), single marital status (*p* = 0.01), no formal education (*p* = 0.022), previous caesarean section (*p* = 0.032), alcohol consumption during pregnancy (*p* = 0.004), and gender-based abuse (*p* = 0.001) were all positively associated with antenatal depression [27].

South Africa has a predominantly patriarchal social structure (male dominance over women in aspects of relationships, political leadership, and social privilege) where high levels of domestic violence occur [53–56]. In South Africa, intimate partner violence is ranked as the second highest burden of disease after HIV/AIDS [56], and the prevalence of femicide is high in this country where 53% of female homicides are committed by intimate partners [57]. Furthermore, South Africa has the highest rate of female homicide as a result of intimate partner violence in the world [58], and the murder rate of South African women is six times the rate of the world average [56, 58, 59]. The current study corroborated this reality as postnatal depression was also associated with physical violence (adjusted odds ratio (aOR) 7.32, 95% CI 1.66-29.48, *p* = 0.005), verbal abuse (adjusted odds ratio (aOR) 4.3, 95% CI 1.03-15.79, *p* = 0.03), and intimate partner violence (adjusted odds ratio (aOR) 9.18, 95% CI 1.58-48.82, *p* = 0.008).

Numerous other studies have documented the positive correlation between intimate partner violence and postpartum depression [46, 54, 60–65], and researchers argue that intimate partner violence has a negative effect on sexual and reproductive health [56]. Because adolescent mothers have already compromised their sexual and reproductive health due to early pregnancy, intimate partner violence compounds the status of their sexual and reproductive health and well-being [55]. The prevalence of intimate partner violence during pregnancy in South Africa is approximately 15.23% [64]. Intimate partner violence is likely to escalate to intimate partner femicide [66]. However, the prevalence of intimate partner violence among our sample of participants was relatively low at 3.7%, which may have been due to underreporting of this form of violence as women are often frightened or embarrassed to report abusive relationships [57]. Many South African women bear the brunt of poverty and often stay in abusive relationships due to economic disempowerment [57], and in this country, many adolescent girls become involved with older men. This increases their vulnerability to power imbalances in the relationship [55, 67] which can fuel abuse [57]. Adolescent women have distorted beliefs about love and abusive behaviour which also contributes to poor disclosure of intimate partner violence [68]. Stressful life events, such as a history of abuse, may result in the resurfacing of internalized emotions. Pregnancy-related changes also lead to negative and harmful thoughts, especially in women with a history of abuse [57]. Therefore, pregnancy and motherhood can be a difficult period for abused women and may increase their susceptibility to depression [69].

## 5. Strengths and Limitations

Our study may add to the knowledge and insight of healthcare providers, researchers, policy makers, and the public as it illuminates various risk factors that impact antenatal and postpartum depression. Moreover, the findings may be used to inform future research and healthcare practices, particularly in instances where pregnant and postpartum adolescents are involved.

However, several limitations impacted the findings. For example, while the results are significant, the confidence intervals are large due to the small sample size and the precise estimates should therefore not be overemphasized. Moreover, a relatively small sample of 326 adolescents who accessed maternal health services at one rural peripheral district hospital was selected, and therefore, the results may not be generalized to all district hospitals in South Africa. A comparison cell of only 38 depressed participants may also not allow for accurate information. A score of 13 or higher on the EPDS scale is suggestive of major depressive disorder. In this regard, by labelling adolescent women with EPDS scores of less than 13 as not depressed, we may have missed those adolescent women with milder depression. Therefore, during screening women with EPDS, it is important for healthcare professionals to classify women according to the severity of the illness. Our study may have found more adolescent women as depressed if the minor depression range were considered. A lower threshold of 9/10 has been recommended by Cox et al. [70] for community screening to ensure that probable cases of depression are identified.

Our cross-sectional study design also limited the findings, and it is suggested that a prospective cohort analysis should be able to yield the true incidence of antenatal and postpartum depression. The cross-sectional design does not allow researchers to establish causality and recall bias, and this fact may also have influenced the results.

## 6. Conclusion

The prevalence of antenatal depression was higher than that of postpartum depression. Physical violence, verbal abuse, and absence of partner support were associated with antenatal and postpartum depression, while intimate partner violence was primarily associated with postpartum depression. The literature has shown that there is much inconsistency regarding the prevalence rates of antenatal and postpartum depression across the globe, and these could be attributed to socioeconomic factors, cultural factors, and health system factors. The literature also suggests that maternal depression and interpersonal violence are under investigation [54], and this study corroborates the need for wider investment in preventative programmes and policies to eradicate violence against women, particularly female adolescents and children in South Africa.

Professionals involved in maternal healthcare need to consider the mental health of pregnant and postpartum adolescents. The World Health Organisation has emphasized “no health without perinatal mental health” [52]. In this regard, mental health needs to be integrated into reproductive health programmes and primary healthcare settings. Furthermore, integrating mental health screening in routine antenatal care will not only ensure early and effective identification of women with mental health problems but also reduce the stigma that is associated with seeking help [71]. However, the training of healthcare professionals in mental health screening is often sidelined, especially in low- and middle-income countries, due to other competing health interests [69]. It is also undeniable that the early identification of antenatal and postnatal depression will improve health outcomes for individuals, infants, partners, and families [37]. Experts in the field of neuropsychology recommend that clinicians in hospitals should conduct universal screening of women for postpartum depression using the EPDS in the first 48 to 72 hours (2-3 days) postdelivery [9, 37, 38]. While some may argue that the in-hospital assessment of mothers postdelivery is too premature for diagnosing postpartum depression, early assessment is a vital opportunity to screen for potential risk factors associated with postnatal depression.

## Figures and Tables

**Table 1:** Summary statistics of numerical variables (*n* = 326).

Variable	Min	^∗^Q25	Median	^∗^Q75	Max	Mean	SD
Age	14	17	18	19	19	17.92	1.2
Number of people contributing to household income	1	1	2	3	5	1.98	0.88

^∗^Q25 and Q75 represent the interquartile range (IQR). Q25 is the 25th percentile. Q75 is the middle value for the last half of the ranked-ordered data. Since the data is not normally distributed, it is usually better to report the medians and IQR, than it is to report the mean and standard deviation.

**Table 2:** Frequency distributions for key categorical variables in this analysis (*n* = 326).

	*n*	%
Highest education level		
Primary	9	2.8
Junior	12	3.7
Secondary	305	93.6
Employed		
No	318	97.5
Yes	8	2.5
Mother's marital status		
Single	147	45.1
Married	101	31
Other	78	23.9
Had abortion		
No	298	91.4
Yes	28	8.6
Repeat pregnancies		
No	261	80.1
Yes	65	19.9
HIV		
No	268	82.2
Yes	58	17.8
Physical violence		
No	306	93.9
Yes	20	6.1
Sexual abuse		
No	312	95.7
Yes	12	3.7
Do not wish to answer	2	0.6
Verbal abuse		
No	289	88.7
Yes	34	10.4
Do not wish to answer	3	0.9
Intimate partner violence		
No	312	95.7
Yes	12	3.7
Refuse to answer	2	0.6
Partner support		
None	23	7.1
Little	17	5.2
Reasonable	120	36.8
A lot	166	50.9
Depression		
Depressed	38	11.7
Not depressed	288	88.3
Pre/postnatal		
Postnatal	194	59.5
Prenatal	132	40.5

**Table 3:** Comparison of the prevalence of depression between pregnant and postpartum adolescents (*n* = 326).

Variables	Depressed *n* = 38	Not depressed *n* = 288	*p* value
Pregnant/postpartum			0.054
Postpartum	17 (44.7%)	177 (61.5%)	
Pregnant	21 (55.3%)	111 (38.5%)	

**Table 4:** Comparison of risk factors for participants who had depression versus those who did not (*n* = 326).

Variables	Depression		
	Depressed No. 38	Not depressed No. 288	*p* value
Age	18.0 (17.0 - 19.0)	18.0 (17.0 - 19.0)	0.81
Number of people contributing to HH income	2.0 (1.0 - 2.0)	2.0 (1.0 - 3.0)	0.86
Highest education level			0.13
Primary	3 (7.9%)	6 (2.1%)	
Junior	1 (2.6%)	11 (3.8%)	
Secondary	34 (89.5%)	271 (94.1%)	
Employed			1.0
No	37 (97.4%)	281 (97.6%)	
Yes	1 (2.6%)	7 (2.4%)	
Mother's marital status			0.63
Single	20 (52.6%)	127 (44.1%)	
Married	10 (26.3%)	91 (31.6%)	
Other	8 (21.1%)	70 (24.3%)	
Had abortion			0.55
No	34 (89.5%)	264 (91.7%)	
Yes	4 (10.5%)	24 (8.3%)	
Repeat pregnancies			0.83
No	30 (78.9%)	231 (80.2%)	
Yes	8 (21.1%)	57 (19.8%)	
HIV			0.37
No	29 (76.3%)	239 (83.0%)	
Yes	9 (23.7%)	49 (17.0%)	
Physical violence			0.0007
No	30 (78.9%)	276 (95.8%)	
Yes	8 (21.1%)	12 (4.2%)	
Sexual abuse			0.010
No	34 (89.5%)	278 (96.5%)	
Yes	2 (5.3%)	10 (3.5%)	
Do not wish to answer	2 (5.3%)	0 (0.0%)	
Verbal abuse			0.002
No	27 (71.1%)	262 (91.0%)	
Yes	11 (28.9%)	23 (8.0%)	
Do not wish to answer	0 (0.0%)	3 (1.0%)	
Intimate partner violence			0.027
No	33 (86.8%)	279 (96.9%)	
Yes	4 (10.5%)	8 (2.8%)	
Refuse to answer	1 (2.6%)	1 (0.3%)	
Partner support			0.018
None	7 (18.4%)	16 (5.6%)	
Little	2 (5.3%)	15 (5.2%)	
Reasonable	16 (42.1%)	104 (36.1%)	
A lot	13 (34.2%)	153 (53.1%)	

All continuous values are reported with median and interquartile range, Med (IQR), while categories are reported in percentages, *n* (%).

**Table 5:** Comparison of risk factors for participants who specifically had depression versus those who did not in the prenatal period (*n* = 132).

Variables	Had depression		
	Not depressed No. 111	Depressed No. 21	*p* value
Age	18.0 (17.0 - 19.0)	19.0 (18.0 - 19.0)	0.39
Number of people contributing to HH income	2.0 (1.0 - 3.0)	2.0 (2.0 - 2.0)	0.83
Highest education level			1.0
Primary	4 (3.6%)	1 (4.8%)	
Junior	7 (6.3%)	1 (4.8%)	
Secondary	100 (90.1%)	19 (90.5%)	
Employed			1.0
No	107 (96.4%)	21 (100.0%)	
Yes	4 (3.6%)	0 (0.0%)	
Mother's marital status			0.75
Single	45 (40.5%)	10 (47.6%)	
Married	37 (33.3%)	7 (33.3%)	
Other	29 (26.1%)	4 (19.0%)	
Had abortion			1.0
No	89 (80.2%)	17 (81.0%)	
Yes	22 (19.8%)	4 (19.0%)	
Repeat pregnancies			0.80
No	74 (66.7%)	15 (71.4%)	
Yes	37 (33.3%)	6 (28.6%)	
HIV			1.0
No	87 (78.4%)	17 (81.0%)	
Yes	24 (21.6%)	4 (19.0%)	
Physical violence			0.036
No	106 (95.5%)	17 (81.0%)	
Yes	5 (4.5%)	4 (19.0%)	
Sexual abuse			1.0
No	104 (93.7%)	18 (85.7%)	
Yes	7 (6.3%)	1 (4.8%)	
Missing	0 (0.0%)	2 (9.5%)	
Verbal abuse			0.007
No	100 (90.1%)	14 (66.7%)	
Yes	10 (9.0%)	7 (33.3%)	
Missing	1 (0.9%)	0 (0.0%)	
Intimate partner violence			0.59
No	106 (95.5%)	20 (95.2%)	
Yes	4 (3.6%)	1 (4.8%)	
Missing	1 (0.9%)	0 (0.0%)	
Partner support			0.095
None	5 (4.5%)	4 (19.0%)	
Little	9 (8.1%)	2 (9.5%)	
Reasonable	37 (33.3%)	7 (33.3%)	
A lot	60 (54.1%)	8 (38.1%)	

**Table 6:** Comparison of risk factors for participants who specifically had depression versus those who did not in the postpartum period (*n* = 194).

Variables	Had depression		
	Not depressed No. 177	Depressed No. 17	*p* value
Age	18.0 (17.0 - 19.0)	18.0 (17.0 - 19.0)	0.47
Number of people contributing to HH income	2.0 (1.0 - 3.0)	2.0 (1.0 - 2.0)	0.55
Highest education level			0.077
Primary	2 (1.1%)	2 (11.8%)	
Junior	4 (2.3%)	0 (0.0%)	
Secondary	171 (96.6%)	15 (88.2%)	
Employed			0.31
No	174 (98.3%)	16 (94.1%)	
Yes	3 (1.7%)	1 (5.9%)	
Mother's marital status			0.58
Single	82 (46.3%)	10 (58.8%)	
Married	54 (30.5%)	3 (17.6%)	
Other	41 (23.2%)	4 (23.5%)	
Had abortion			1.00
No	175 (98.9%)	17 (100.0%)	
Yes	2 (1.1%)	0 (0.0%)	
Repeat pregnancies			1.0
No	157 (88.7%)	15 (88.2%)	
Yes	20 (11.3%)	2 (11.8%)	
HIV			0.15
No	152 (85.9%)	12 (70.6%)	
Yes	25 (14.1%)	5 (29.4%)	
Physical violence			0.009
No	170 (96.0%)	13 (76.5%)	
Yes	7 (4.0%)	4 (23.5%)	
Sexual abuse			0.31
No	174 (98.3%)	16 (94.1%)	
Yes	3 (1.7%)	1 (5.9%)	
Verbal abuse			0.049
No	162 (91.5%)	13 (76.5%)	
Yes	13 (7.3%)	4 (23.5%)	
Missing	2 (1.1%)	0 (0.0%)	
Intimate partner violence			0.013
No	173 (97.7%)	13 (76.5%)	
Yes	4 (2.3%)	3 (17.6%)	
Missing	0 (0.0%)	1 (5.9%)	
Partner support			0.10
None	11 (6.2%)	3 (17.6%)	
Little	6 (3.4%)	0 (0.0%)	
Reasonable	67 (37.9%)	9 (52.9%)	
A lot	93 (52.5%)	5 (29.4%)	

All continuous values are reported with median and interquartile range, Med (IQR), while categories are reported in percentages, *n* (%).

**Table 7:** Univariable and multivariable logistic regression analysis investigating the influence of selected variables on depression during the antenatal period.

Variable	Univariable			Multivariable		
	Odds ratio	95% CI of the odds ratio	*p* value	Adjusted odds ratio (aOR)	95% CI of the aOR	*p* value
Physical violence	4.99	1.14–20.75	0.0256	6.47	1.36–30.53	0.0160
Sexual abuse	0.83	0.04–5.04	0.8614	0.89	0.05–5.68	0.9184
Verbal abuse	5	1.59–15.31	0.0047	4.82	1.5–15.16	0.0069
Intimate partner violence	1.33	0.07–9.55	0.8057	1.17	0.06–8.75	0.8917
Partner support:						
(1) Reasonable support	0.24	0.05–1.15	0.0670	0.13	0.02–0.76	0.0244
(2) A lot of support	0.17	0.04–0.79	0.0198	0.07	0.01–0.45	0.0050

CI: confidence interval. The multivariable model adjusted for age, HIV status, and the number of people in the household contributing to income.

**Table 8:** Univariable and multivariable logistic regression analysis investigating the influence of selected variables on depression during the postpartum period.

Variable	Univariable			Multivariable		
	Odds ratio	95% CI of the odds ratio	*p* value	Adjusted odds ratio (aOR)	95% CI of the aOR	*p* value
Physical violence	7.47	1.77–28.33	0.0035	7.32	1.66–29.48	0.0055
Sexual abuse	3.62	0.17–30.24	0.2767	2.43	0.1–24.75	0.4867
Verbal abuse	3.83	0.98–12.75	0.0358	4.3	1.03–15.79	0.0322
Intimate partner violence	9.98	1.81–50.18	0.0048	9.18	1.58–48.82	0.0087
Partner support:						
(1) reasonable support	0.49	0.12–2.47	0.3397	0.59	0.14–3.13	0.4951
(2) A lot of support	0.2	0.04–1.06	0.0416	0.23	0.05–1.3	0.0733

CI: confidence interval. The multivariable model adjusted for age, HIV status, and the number of people in the household contributing to income.

## Data Availability

The data that were used to elicit the findings of the study are available from the corresponding author (DG).
